# A Single-Institution Comparative Analysis of Bracketed Localization for Partial Mastectomy Using Traditional Wire and Wireless Methods

**DOI:** 10.1245/s10434-026-19959-6

**Published:** 2026-07-06

**Authors:** Olivia W. Galloway, Ashley Wilbers, Omolade O. Sogade, Ariana Naaseh, Ami Trivedi, Justin Dumrongkulraksa, Jade Tao, Jingqin Luo, Arianna Buckley, Julie A. Margenthaler

**Affiliations:** 1 Division of Surgical Oncology, Department of Surgery, Washington University in St. Louis School of Medicine, St. Louis, MO USA; 2 Department of Surgery, University of Missouri School of Medicine, Columbia, MO USA; 3 Department of Breast Imaging, Mallinckrodt Institute of Radiology, Washington University in St. Louis School of Medicine, St. Louis, MO USA; 4 Siteman Cancer Center Biostatistics Shared Resource, Department of Surgery, Washington University in St. Louis School of Medicine, St. Louis, MO USA

**Keywords:** Bracketed localization, Breast, Wire localization, Magnetic seed localization

## Abstract

**Background:**

The use of multiple wires or wireless seeds to bracket large areas of breast lesions is an effective way to avoid mastectomy in patients. However, direct comparisons between wire and wireless bracketed localization are not well studied. We aimed to compare wireless and wire bracketed localization and identify factors associated with positive or close margins.

**Methods:**

Patients undergoing bracketed localization for malignancy from 2019 to 2024 were identified. Data collected included demographics, radiographic features, pathology, and margin status. Patients with benign pathology and those undergoing oncoplastic surgery were excluded. Characteristics were compared between patient groups by localization type and margin status.

**Results:**

In total, 240 patients were identified. Of these, 175 (72.9%) underwent wire bracketed localization and 65 (27.1%) underwent wireless bracketed localization. There were no significant differences in final margin status, distance from the closest margin, or need for additional surgery. A total of 66 patients (28.5% of wire bracketed localization and 24.6% of wireless bracketed localization) had close or positive margins, which was associated with size of disease, grade, atypical ductal hyperplasia on core biopsy, ductal carcinoma in situ or invasive lobular carcinoma on final pathology, number of biopsy sites, and a lack of discrete mass or calcifications on imaging.

**Conclusions:**

In this large single-institution study, wireless and wire bracketed localization had similar final margin status. Although we could not control completely for selection bias between methods, we were able to demonstrate factors associated with positive or close margins to determine scenarios in which wider margins or additional surgery may be necessary.

**Supplementary Information:**

The online version contains supplementary material available at 10.1245/s10434-026-19959-6.

The surgical options for breast cancer include breast-conservation therapy, which involves a partial mastectomy and adjuvant radiation therapy, or mastectomy. Both of these procedures have similar oncologic outcomes.^1,2^ An important component of breast conservation is obtaining negative surgical margins, defined as no ink on tumor for invasive carcinoma or margins >2 mm for ductal carcinoma in situ (DCIS).^3,4^ Women with large cancers who will have adequate residual breast volume after excision may still be offered partial mastectomy, but they may still have a higher risk for positive margins requiring additional surgery.^5^

In 1987, Silverstein et al.^6^ introduced the concept of using multiple wires to bracket large areas of calcifications. Since that initial report, multiple studies have shown that bracketed wire localization to mark the extent of large areas of disease on imaging can ensure complete removal of calcifications, allow for more optimal resection volumes, and decrease the number of procedures required to obtain clear margins.^7–12^ However, wire localization has several considerations, such as the need for same-day insertion requiring coordination between the operating room and radiology department, possible wire migration, the potential for delays in operating room start times, and nonoptimal sites of wire insertion. Wireless localization utilizing radioactive seeds, radar reflectors, radiofrequency identification tags, or magnetic seeds allow for the uncoupling of the operating room and radiology department and fewer operating room delays (Fig. 1).^13^

**Fig. 1:**
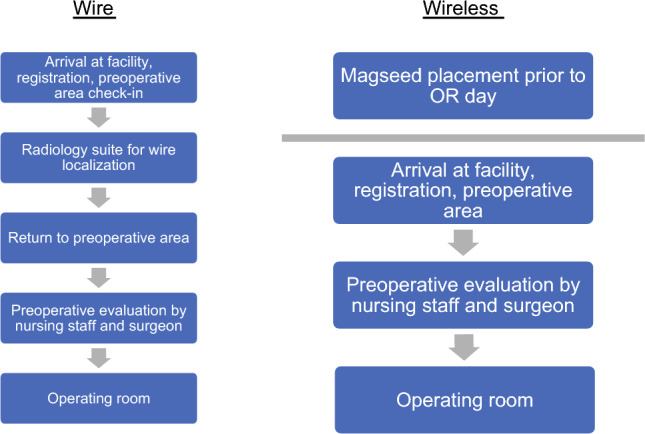
Schematic of workflow for wire bracketed localization and wireless bracketed localization

Previous studies have found that magnetic seed localization and wire localization are similarly effective for the localization and removal of breast lesions, but these studies did not specifically investigate rates of re-excision or margin positivity in bracketed localization.^14,15^ Bracketed localization utilizing other wireless modalities such as radioactive seeds^13,16–19^ and radar reflectors^13,20^ have been previously investigated and found to be feasible and oncologically safe, with negative margins achieved in 70–80% of patients. Only one study in the literature has directly compared radioactive seed bracketed localization with wire bracketed localization, reporting that patients were more likely to undergo re-excision for close or positive margins with wire bracketed localization.^17^ This study did not evaluate magnetic seed (Magseed) bracketed localization, and no studies have compared magnetic seed bracketed localization and wire bracketed localization. Several studies have shown that the presence of DCIS, multifocality, and invasive lobular carcinoma (ILC) have been correlated with positive margins with bracketed wire or radioactive seed localization, though these studies were small single-institution studies that consisted of 113 and 157 cases, respectively, and did not compare localization modalities.^16,20^

This single-institution retrospective review aimed to compare rates of positive margins and re-excision in wire bracketed localization and magnetic seed bracketed localization in a larger cohort of patients. Additionally, we aimed to identify any clinicopathologic characteristics that predicted close or positive margins in bracketed localization.

## Methods

After institutional review board approval, the records of all patients who had multiple wires or magnetic seeds placed for bracketed localization for malignancy with subsequent partial mastectomy at Washington University in St. Louis between 2019 and 2024 were reviewed. Patients who were undergoing resection of multiple sites of disease requiring multiple localization devices to perform multiple lumpectomies, and were therefore not truly bracketed localizations, and patients who had undergone oncoplastic surgery were excluded. The decision to use each localization method was at the discretion of the breast surgeon in combination with a breast-dedicated radiologist.

A retrospective chart review of these cases was performed. Demographic data, including age, sex, race, ethnicity, menopausal status, family history, genetic testing, and body mass index (BMI) were collected. Radiographic features, including breast density, method of detection, imaging findings, Breast Imaging Reporting and Data Systemclassification, and largest extent of disease were recorded and analyzed. Operative findings such as collection of shave margins, the localization modality, and device with wires or Magseed (Hologic, Marlborough, MA, USA) were recorded and analyzed. The Magseed is a 5 mm metallic magnetic marker that was approved by the US Food and Drug Administration in 2016. The sensing depth of the Magseed is 40 mm, and two seeds can be bracketed and sensed when the separation between seeds is ≥20 mm. It is not radioactive and cannot be inactivated by electrocautery. The Magseed was used for all wireless localizations.

The decision to obtain shave margins at the time of the initial surgery was determined by the breast surgeon based on a combination of specimen radiograph findings, input from the breast radiologist, and intraoperative findings. Pathology findings on core biopsy, final pathology findings, and the final margin status after initial surgery, size of specimen, the final size of disease, T stage, and shave margins were recorded and analyzed. In accordance with national guidelines,^21^ negative margins were achieved if there was no tumor on ink for invasive carcinoma and ≥2 mm for DCIS. Close margins were defined as <2 mm for DCIS. In our analysis, margins for invasive carcinoma were defined as positive or negative in accordance with national guidelines. Close margins for invasive carcinoma were not considered. Positive margins for DCIS and invasive carcinoma and close margins for DCIS were analyzed together. The number and type of additional surgeries were collected.

### Statistical Analysis

Patient characteristics were compared using the χ2 test or Fisher’s exact test for categorical variables, and Student’s t-test was used for continuous variables between wire and wireless localization or between no additional surgery and additional surgery. Patients with a pathologic complete response were excluded from the analysis of factors influencing positive or close margin status. A multivariable Firth logistic regression model was applied to identify risk factors for positive or close margins, and the backward selection method was applied to select significant covariates in the model. Firth logistic regression analysis results are expressed as odds ratios, 95% confidence intervals (CIs), and *p* value. The post hoc power of the study to compare the rate of positive/close final margins using a 2-sided two sample proportion test (based on normal approximation) at a 5% significance level was calculated.

All tests were two-sided, and a *p*-value ≤0.05 was considered statistically significant. Statistical analyses were performed within SAS (version 9.4; SAS Institute, Cary, NC, USA).

## Results

A total of 240 patients were identified, of whom 175 (72.9%) had received wire bracketed localization and 65 (27.1%) wireless bracketed localization. The average age was 62.86 years, and patients in the wireless group were slightly older, with an average age of 65.29 years compared with the wire group, which had an average age of 61.97 years (*p* = 0.032, Table 1). Patients in the wireless group had a significantly lower BMI than those in the wire group (29.14 vs. 32.8, *p* < 0.001, Table 1). In total, 80% of patients in the wireless group and 67.71% in the wire group were white (*p* = 0.047). There were no significant differences in menopausal status, family history, presence of a genetic mutation, or use of hormone replacement between groups. There were no significant differences in imaging findings, such as architectural distortion, calcifications, focal asymmetry, mass, or non-mass enhancement, and breast density between the wire and wireless groups (Table 2). The median number of localization devices in each group was two, with a range of two to four in the wire group and a range of two to three in the wireless group (Table 2).

**Table 1:** Comparison of patient demographics between wire and wireless localization

Variable	Level	Wire	Wireless	*P*-value
		*N* = 175	*N* = 65	
Age at diagnosis		61.97 (10.63)	65.29 (10.48)	**0.032**
Race	Black	56 (32.00%)	11 (16.92%)	**0.047**
	White	115 (65.71%)	52 (80.00%)	
	Other	4 (2.29%)	2 (3.08%)	
BMI		32.80 (7.76)	29.14 (6.04)	**<.001**
Menopausal status	Pre-menopausal	28 (16.00%)	6 (9.23%)	0.051
	Post-menopausal	147 (84.00%)	57 (87.69%)	
	Hysterectomy	0 (0.00%)	1 (1.54%)	
	Uncertain	0 (0.00%)	1 (1.54%)	
Family history	No	94 (53.71%)	26 (40.00%)	0.059
	Yes	81 (46.29%)	39 (60.00%)	
Genetic mutation	BRCA 1	0 (0.00%)	1 (1.85%)	0.512
	BRCA2	1 (0.76%)	0 (0.00%)	
	CHEK2	1 (0.76%)	1 (1.85%)	
	FANCC	1 (0.76%)	0 (0.00%)	
	PALB2	1 (0.76%)	0 (0.00%)	
	Other	1 (0.76%)	1 (1.85%)	
	None	127 (96.21%)	51 (94.44%)	
Hormone replacement	Current	3 (1.71%)	3 (4.62%)	0.301
	Former	28 (16.00%)	13 (20.00%)	
	Never	140 (80.00%)	49 (75.38%)	
	Unknown	4 (2.29%)	0 (0.00%)	

Data are presented as *n* (%) unless otherwise indicated. Bolded *p*-values less than 0.05 are considered significant

BMI, body mass index

**Table 2:** Comparison of clinicopathological characteristics and surgical outcomes between wire and wireless bracketed localization

Variable	Level	Wire	Wireless	*P*-value
		*N* = 175	*N* = 65	
Number of localization devices		2 (2-4)	2 (2-3)	0.21
Extent of disease on imaging (cm)		4.11 (2.12)	3.26 (1.41)	**<.001**
Size of specimen (cm)		7.53 (2.21)	7.24 (1.89)	0.354
Final margin status	Positive/Close	50 (28.57%)	16 (24.41%)	0.854
	Negative	125 (71.42%)	49 (75.38%)	
Final Pathology	IDC	76 (43.43%)	32 (49.23%)	0.416
	ILC	19 (10.86%)	2 (3.08%)	
	DCIS only	60 (34.29%)	24 (36.92%)	
	Other	7 (4.00%)	2 (3.08%)	
	pCR	13 (7.43%)	5 (7.69%)	
ER/PR/HER2 status	DCIS ER+	55 (31.43%)	24 (36.92%)	0.703
	DCIS ER-	12 (6.86%)	6 (9.23%)	
	HER2+	18 (10.29%)	7 (10.77%)	
	HR+/HER2-	77 (44.00%)	22 (33.85%)	
	Triple negative	13 (7.43%)	6 (9.23%)	
Closest margin (mm)		2.23 (2.32)	2.64 (2.58)	0.263
Additional surgery	No	128 (73.14%)	48 (73.85%)	0.913
	Yes	47 (26.86%)	17 (26.15%)	
Number of additional surgeries	0	61 (56.48%)	20 (54.05%)	0.962
	1	41 (37.96%)	15 (40.54%)	
	2	5 (4.63%)	2 (5.41%)	
	3	1 (0.93%)	0 (0.00%)	
Shave margins	No	104 (60.47%)	37 (56.92%)	0.62
	Yes	68 (39.53%)	28 (43.08%)	
Breast Density	Fatty	11 (6.29%)	1 (1.54%)	0.376
	Scattered	76 (43.43%)	32 (49.23%)	
	Heterogenous	80 (45.71%)	28 (43.08%)	
	Extremely dense	7 (4%)	4 (6.15%)	
Architectural distortion	No	157 (89.71%)	58 (89.23%)	0.913
	Yes	18 (10.29%)	7 (10.77%)	
Calcifications	No	74 (42.29%)	26 (40%)	0.75
	Yes	101 (57.71%)	39 (60%)	
Focal asymmetry	No	149 (85.14%)	57 (87.69%)	0.615
	Yes	26 (14.86%)	8 (12.31%)	
Mass	No	95 (54.29%)	43 (66.15%)	0.098
	Yes	80 (45.71%)	22 (33.85%)	
Nonmass enhancement	No	155 (88.57%)	62 (95.38%)	0.141
	Yes	20 (11.43%)	3 (4.62%)	
Final T stage	T0	14 (8%)	5 (7.69%)	0.395
	T1	78 (44.57%)	32 (49.23%)	
	T2	22 (12.57%)	3 (4.62%)	
	T3	3 (1.71%)	2 (3.08%)	
	Tis	56 (32%)	23 (35.38%)	
Final size of disease (cm)		2.16 (1.78)	2.04 (1.72)	0.63

Data are presented as n (%) unless otherwise indicated. Bolded *p*-values less than 0.05 are considered significant

DCIS, ductal carcinoma in situ; ER, estrogen receptor; HER2, human epidermal growth factor receptor 2; HR, hormone receptor; IDC, invasive ductal carcinoma; ILC, invasive lobular carcinoma; pCR, pathological carcinoma

Patients in the wireless group had a significantly smaller extent of disease on imaging than those in the wire group (3.26 vs. 4.11 cm, *p* <0.001, Table 2). There was no difference in size of the specimen, final margin status, distance from the closest margin, shave margins, need for additional surgery, or number of additional surgeries between the wire and wireless groups (Table 2). Final T stage, size of disease, and final pathologic findings were similar between the wire and wireless groups (Table 2).

In total, 66 patients (27.5%) had close or positive margins and 64 patients underwent additional surgery. Preoperative breast magnetic resonance imaging (MRI) was not significantly associated with margin status (*p* = 0.3330) or re-excision rates (*p* = 0.816). Among those with preoperative MRI, 29.03% had positive or close margins (95% CI 22.62–37.53), whereas 23.52% of patients without preoperative MRI had positive or close margins (95% CI 15.00–33.97). There was no significant association between specimen radiograph findings and re-excision rates. Re-excision rates were estimated to be 36.36%, (12/33 [95% CI 20.40–54.88]), 27.27% (3/11 [95% CI 6.02–60.97]), 25.14% (46/183 [19.03–32.07]), and 25% (3/12 [95% CI 5.49–57.19]) for calcifications at edge of specimen, clip not retrieved, lesion/calcifications and clips removed entirely, and lesion at edge of specimen respectively (*p* = 0.612). Surgeons were more likely to take additional shave margins at the time of surgery if the specimen radiograph showed calcifications at edge of specimen, the lesion at edge of specimen, or if the clip was not retrieved than if the specimen radiograph showed the lesion or calcifications with clips removed in their entirety (62.96% vs. 37.04%, *p*<0.001).

There were no differences in demographic features between patients with positive/close margins and those with negative margins. On univariate analysis, the extent of disease on preoperative imaging was associated with margin status (Table 3). The largest extent of disease on imaging was 4.25 cm in those with close or positive margins and 3.62 cm in those with negative margins (*p* = 0.019, Table 3). T stage and size of disease on pathology were also associated with margin status (Table 3). Of patients that had positive or close margins, 6.25% had T3 disease, whereas 0.65% of those with negative margins had T3 disease (*p*<0.001, Table 3). Those with positive or close margins had an average of 3.24 cm of disease on final pathology compared with 1.92 cm in those with negative margins (*p*<0.001, Table 3). Additionally, on final pathology, DCIS and ILC were associated with positive or close margins, and 54.55% and 7.58% of patients with positive or close margins had DCIS and ILC, respectively, compared with 30.32% and 5.16% of patients with negative margins, respectively (*p* = 0.004, Table 3). Grade 3 disease was more likely to be associated with positive or close margins (*p* = 0.042), as was the presence of atypical ductal hyperplasia (ADH) on core needle biopsy (*p* = 0.028, Table 3). The only imaging finding associated with positive or close margins was a lack of mass identified on preoperative imaging (*p* = 0.029, Table 3).

**Table 3:** Comparison of factors associated with positive or close margins and negative margins

Variable	Level	Negative	Pos/close	*P*-value
		*N* = 155	*N* = 66	
Largest extent of disease (cm)		3.62 (1.73)	4.25 (1.93)	**0.019**
Size of specimen (cm)		7.53 (1.96)	7.35 (2.59)	0.615
Final Pathology	DCIS only	47 (30.32%)	36 (54.55%)	**0.004**
	IDC	38 (24.52%)	5 (7.58%)	
	IDC with DCIS	50 (32.26%)	15 (22.73%)	
	ILC	8 (5.16%)	5 (7.58%)	
	ILC with DCIS	5 (3.23%)	2 (3.03%)	
	Other	7 (4.52%)	3 (4.55%)	
ER/PR/HER2 status	DCIS ER+	46 (29.68%)	33 (50.00%)	**0.011**
	DCIS ER-	10 (6.45%)	8 (12.12%)	
	HER2+	11 (7.10%)	2 (3.03%)	
	HR+/HER2-	78 (50.32%)	21 (31.82%)	
	Triple negative	10 (6.45%)	2 (3.03%)	
Shave margins	No	85 (55.19%)	40 (62.50%)	0.321
	Yes	69 (44.81%)	24 (37.50%)	
Number of biopsy sites	1	111 (71.61%)	43 (66.15%)	0.118
	2	34 (21.94%)	21 (32.31%)	
	3	10 (6.45%)	1 (1.54%)	
ADH on core	No	149 (96.75%)	59 (89.39%)	**0.028**
	Yes	5 (3.25%)	7 (10.61%)	
LCIS on core	No	147 (95.45%)	62 (93.94%)	0.737
	Yes	7 (4.55%)	4 (6.06%)	
ALH on core	No	150 (98.04%)	66 (100.00%)	0.556
	Yes	3 (1.96%)	0 (0.00%)	
Grade	I	38 (25.00%)	8 (12.50%)	**0.042**
	II	70 (46.05%)	28 (43.75%)	
	III	44 (28.95%)	28 (43.75%)	
Final T stage	T1	91 (58.71%)	19 (29.69%)	**<.001**
	T2	18 (11.61%)	7 (10.94%)	
	T3	1 (0.65%)	4 (6.25%)	
	Tis	45 (29.03%)	34 (53.13%)	
Number of foci	1	111 (81.02%)	41 (78.85%)	0.487
	2	20 (14.60%)	11 (21.15%)	
	3	3 (2.19%)	0 (0.00%)	
	5	3 (2.19%)	0 (0.00%)	
Architectural distortion	No	141 (90.97%)	59 (89.39%)	0.715
	Yes	14 (9.03%)	7 (10.61%)	
Calcifications	No	66 (42.58%)	25 (37.88%)	0.516
	Yes	89 (57.42%)	41 (62.12%)	
Focal asymmetry	No	134 (86.45%)	55 (83.33%)	0.547
	Yes	21 (13.55%)	11 (16.67%)	
Mass	No	86 (55.48%)	47 (71.21%)	**0.029**
	Yes	69 (44.52%)	19 (28.79%)	
Nonmass enhancement	No	142 (91.61%)	58 (87.88%)	0.386
	Yes	13 (8.39%)	8 (12.12%)	
Final size of disease (cm)		1.92 (1.47)	3.24 (1.89)	**<.001**

Bolded *p*-values less than 0.05 are considered significant

ADH, atypical ductal hyperplasia; ALH, atypical lobular hyperplasia; DCIS, ductal carcinoma in situ; ER, estrogen receptor; HER2, human epidermal growth factor receptor 2 ; HR, hormone receptor; IDC, invasive ductal carcinoma; ILC, invasive lobular carcinoma; LCIS, lobular carcinoma in situ; pCR, pathological carcinoma; PR, progesterone receptor

On multivariable analysis, the type of localization device was again not associated with final margin status (OR 0.873 [95% CI 0.398–1.91], *p* = 0.735, Table 4). The presence of two biopsy sites was associated with an approximately 3-fold higher chance of positive/close margins than was the presence of one biopsy site (OR 3.09 [95% CI 1.4–6.82], *p* = 0.005, Table 4). Patients with grade 3 disease have approximately 2.3 times the risk of positive/close margins compared with those with grade 2 disease (OR 2.27 [95% CI 1.05–4.9], *p* = 0.038, Table 4). Lack of calcifications and lack of mass on preoperative imaging were associated with positive/close margins (OR 2.56 [95% CI 1.05–6.26], *p* = 0.037 and OR 2.46 [95% CI 0.998–6.06], *p* = 0.051, respectively, Table 4). The final size of disease on pathology was associated with close/positive margins (OR 1.52 [95% CI 1.23–1.88], *p*<0.001), but size of disease on preoperative imaging was not (*p* = 0.141, Table 4).

**Table 4:** Multivariable analysis of factors associated with positive or close margins

Effect	*P* value	Odds ratio	95% CI	
Localization device	0.735	0.873	0.398	1.91
Wire versus Wireless				
Family history	0.288	1.44	0.736	2.81
No versus Yes				
Number of biopsy sites	**0.019**			
2 versus 1	0.005	3.09	1.4	6.82
3 versus 1	0.952	1.06	0.158	7.12
Grade	0.066			
I versus III	0.061	0.373	0.133	1.05
III versus II	**0.038**	2.27	1.05	4.9
Largest extent of disease on preoperative imaging	0.141	1.16	0.95	1.4
Calcifications	**0.039**	2.56	1.05	6.26
No versus Yes				
Mass	**0.051**	2.46	0.998	6.06
No versus Yes				
Final size of disease	**<0.001**	1.52	1.23	1.88

Bolded *p*-values less than 0.05 are considered significant

CI, confidence interval

## Discussion

In our large single-institution study, we compared outcomes between wire and wireless bracketed localization. Within our cohort, there were no differences in rate of positive or close margins, need for additional surgery, or number of additional surgeries by type of localization method. On multivariable analysis, there was no impact of type of localization on margin status. Additionally, size of disease on imaging and final pathology, T stage, grade, DCIS on final pathology, and ADH on core needle biopsy were associated with positive or close margins on univariate analysis. On multivariable analysis, two biopsy sites were three times as likely to have a close or positive margin than a single biopsy site, grade 3 disease was twice as likely to have a close or positive margin as grade 2 disease, a lack of calcifications or mass on imaging was approximately 2.5 times more likely to have a positive margin, and final size of disease was associated with positive or close margins.

In the study by Da Silva et al.^17^ comparing radioactive seed bracketed localization and wire bracketed localization, patients who underwent wire bracketed localization were 3.9 times more likely to undergo re-excision than patients who underwent radioactive seed bracketed localization. However, patients in the seed group were more likely to undergo oncoplastic procedures, which have been shown to have lower rates of positive margins and re-excision.^22^ To eliminate this potential source of confounding, we excluded patients with oncoplastic procedures from our study, and there was no difference in margin status or rate of additional surgeries in this study.

In this study, the positive and close margin rate was 27.5% among all patients, 28.6% in the wire group, and 24.6% in the wireless group. Positive and close margin rate in bracketed localization has been reported to be 14–35% in previous studies evaluating bracketed localization utilizing wires, radioactive seeds, or SAVI Scout.^7,12,16–18,20^ This study is the largest to compare positive and close margin rates when utilizing magnetic seed bracketed localization versus wire bracketed localization. In addition to the advantages provided by the wireless nature of localization such as reduction in complexity of scheduling leading to decreased operating room delays and patient time spent in the hospital and preoperative area,^13^ the Magseed has multiple advantages over other wireless technologies, such as a lack of radioactivity, no deactivation when in contact with electrocautery, and the ability to be used in the presence of a hematoma, allowing for an additional wireless modality to be utilized in bracketed localization with similar surgical outcomes. Wireless localization also has advantages with regards to the patient experience. Patients have reported less pain and anxiety and more convenience with wireless localization than with wireless localization.^23,24^ Radiologists and surgeons have also rated Magseed as an easier localization procedure than wire localization and as easier to localize intraoperatively.^24^ Although Magseeds are more expensive than wires, the ability to uncouple localization from operating room time enables surgeons to perform more first-start operating room cases and more cases as a result of fewer operating room delays. The national average Medicare rate for facility payments for partial mastectomies is $US3,829.28,^25^ whereas a Magseed costs about $US250. Therefore, it is cost effective to use Magseeds in settings where at least one additional case per day is feasible. Lastly, although the adoption of any new technology requires additional institutional training and resources, the adoption of the Magseed was seamless at our institution. The decision to change to a wireless system was a collaboration between surgeons and radiologists after a consideration of the above costs, and operating room and radiology suite support was provided by the Hologic clinical team for the first 2 weeks to ensure all staff members were comfortable with the new system. Given these advantages with similar re-excision rates, the Magseed is an excellent alternative to wire bracketed localization in this population. One disadvantage of the Magseed is that it causes MRI artifacts. At our institution, they are only placed after MRI evaluation is complete so as not to interfere with MRIs to assess neoadjuvant treatment response. This limitation will need to be considered at any institution implementing this specific wireless system. From the patient’s perspective, this may involve an additional procedure for Magseed placement in comparison with other wireless modalities that do not interfere with MRIs and may be placed at the time of biopsy. However, the number of procedures is equivalent between Magseed and wire localization. Lastly, additional analysis regarding specimen volumes and weights is required to fully compare the two techniques. Although there was no difference in measured size of the specimen in its greatest dimension between wireless and wire bracketed localization, there was no comparison of specimen weight or volume. Our study did not analyze differences in localization times, operative times, cosmetic outcomes, or specimen weights or volumes, which will be a subject for further investigation to directly compare the practical implications and surgical quality assessment between these two localization modalities.

This study also sought to identify clinicopathologic characteristics associated with positive or close margin status. On univariate analysis, the presence of DCIS or ILC on final pathology, larger size of disease on preoperative imaging and final pathology, and T3 disease were associated with positive or close margins, similar to previously reported data.^16,20^ Patients with positive margins had an average of 4.25 cm of disease on preoperative imaging compared with 3.62 cm in those with negative margins. Additional factors associated with positive or close margins in this study included higher grade of disease, a lack of discrete mass or calcifications on preoperative imaging, and presence of ADH on core biopsy. These associations can be used to help counsel patients regarding their increased likelihood for positive margins or need for additional surgery and may influence patients’ decisions to undergo breast conservation or mastectomy. The need for re-excision has been associated with dissatisfaction with the decision to undergo lumpectomy and a negative impact on patient well-being.^26^ The identification of these specific risk factors for re-excision in this cohort of patients undergoing bracketed localization will allow for a more comprehensive preoperative counseling visit.

This study is limited by its retrospective, single-institution nature. Its retrospective nature meant we could not evaluate the factors that influenced patient and surgeon decisions for bracketed localization versus mastectomy. Several baseline characteristics also differed between the wire and wireless groups, such as a slightly older age, lower BMI, and fewer Black patients in the wireless group. These factors did not influence margin status or the need for additional surgery but may have affected surgeon or patient decisions to undergo wire or wireless localization, which could not be evaluated in this study. There was a slightly larger span of disease on imaging in the wire group than in the wireless group, which may be a potential source of selection bias. At our institution, each surgeon preferentially uses one localization modality for their bracketed localizations and generally do not take size on imaging into account when choosing localization modality, limiting possible selection bias. Each surgeon only utilizes one modality for all their bracketed localizations, and they do not switch between modalities regardless of span of disease or other factors. Our results may only be applicable to surgeons who are already facile with a particular localization modality. Additionally, in our logistic regression model, we adjusted for the extent of disease on imaging, which mitigates selection bias. The estimated ORs were sometimes accompanied with wide CIs, mostly due to the low frequencies of some subgroups (e.g., ILC, DCIS) even after the application of Firth logistic regression, suggesting that our data set may still be underpowered to render precise estimates to such ORs. Another potential source of selection bias in this study is in the decision to order preoperative MRI, which has been shown to decrease the rates of positive margins. Although MRI was not significantly associated with margin status, MRI was ordered at the discretion of each surgeon, and reasons for ordering MRI could not be evaluated in this study. Lastly, there was only 8.9% power to detect the small 4% difference in the rate of positive or close margins, and a total sample size of 3830 would be needed to detect this small difference of 4% with 80% power using a 2-sided two-sample proportion test (based on normal approximation at a 5% significance level), and only very large clinical trials would have sufficient power to detect this difference to determine true noninferiority. This study does show that, at our institution, among surgeons who utilize either wireless or wire localization devices to bracket large or multiple lesions, there is a very small difference in rates of positive or close margins.

Unlike other studies, patients undergoing oncoplastic surgery were excluded from this study. Our positive and close margin rate is within the higher end of the ranges of those reported in prior studies, but this is probably because we excluded oncoplastic surgery and likely would have been reduced if patients undergoing oncoplastic surgery had been included. In this study, all specimens were intraoperatively radiographically examined, and specimens not reported to have the entirety of the lesion, calcifications, or mass present had additional shave margins taken at the time of their index operation.

Lastly, long-term follow-up of survival and recurrence were not investigated in this study. Prospective studies with larger sample sizes and longer follow-up are required to better evaluate the decision-making rationale for type of localization and to assess long-term oncologic outcomes. Long-term oncologic equivalence between wire bracketed localization and magnetic seed bracketed localization therefore cannot be concluded from this data set.

## Conclusion

In this single-institution analysis of bracketed localization in patients with breast cancer who did not undergo oncoplastic procedures, there were no differences in margin status or need for additional surgery between wire and wireless localization. Given the advantages of wireless localization with regards to patient comfort, ease of operating room scheduling and workflow, and cost effectiveness resulting from improved operating room efficiency, wireless bracketed localization for large areas of disease may be used with equivalent rates of positive or close margins and need for re-excision when compared with wire bracketed localization. Positive or close margins were associated with size of disease, high grade of disease, a lack of discrete mass or calcifications on imaging, the presence of ILC or DCIS on final pathology, multiple biopsy sites, and ADH on core needle biopsy, which are important factors to discuss with patients who desire breast-conserving surgery but may be at higher risk for additional surgeries to ensure negative margins, even when bracketed localization is used. 

## Supplementary Information

Below is the link to the electronic supplementary material.

Supplementary file1 (DOCX 19 KB)
