# Supplementary material for: A Single-Institution Comparative Analysis of Bracketed Localization for Partial Mastectomy Using Traditional Wire and Wireless Methods
Source: Ann Surg Oncol. 2026 Jul 6;33(9):8216–24. doi: 10.1245/s10434-026-19959-6 (PMC13452878; doi:10.1245/s10434-026-19959-6)
Supplement: Supplementary file 1 [file 10434_2026_19959_MOESM1_ESM.docx]

**Supplemental Table A.** Demographic characteristics between patients with negative margins and those with positive or close margins.

| **Variable** | **Level** | **Negative** | **Pos/close** | **P-Value** |
| --- | --- | --- | --- | --- |
|  |  | **N=155** | **N=66** |  |
| Age at diagnosis |  | 64.01 (10.68) | 62.73 (9.38) | 0.4 |
| Race | Black | 38 (24.52%) | 23 (34.85%) | 0.246 |
|  | White | 113 (72.90%) | 41 (62.12%) |  |
|  | Other | 4 (2.58%) | 2 (3.03%) |  |
| BMI |  | 31.94 (7.34) | 31.75 (7.34) | 0.863 |
| Menopausal status | Pre-menopausal | 20 (12.90%) | 6 (9.09%) | 0.353 |
|  | Post-menopausal | 134 (86.45%) | 59 (89.39%) |  |
|  | Hysterectomy | 0 (0.00%) | 1 (1.52%) |  |
|  | Uncertain | 1 (0.65%) | 0 (0.00%) |  |
| Family history | No | 70 (45.16%) | 39 (59.09%) | 0.058 |
|  | Yes | 85 (54.84%) | 27 (40.91%) |  |
| Genetic mutation | BRCA 1 | 1 (0.85%) | 0 (0.00%) | 0.918 |
|  | BRCA2 | 1 (0.85%) | 0 (0.00%) |  |
|  | CHEK2 | 1 (0.85%) | 1 (1.79%) |  |
|  | FANCC | 1 (0.85%) | 0 (0.00%) |  |
|  | PALB2 | 1 (0.85%) | 0 (0.00%) |  |
|  | Other | 1 (0.85%) | 1 (1.79%) |  |
|  | None | 111 (94.87%) | 54 (96.43%) |  |
| Hormone replacement | Current | 3 (1.94%) | 2 (3.03%) | 0.741 |
|  | Former | 27 (17.42%) | 10 (15.15%) |  |
|  | Never | 122 (78.71%) | 54 (81.82%) |  |
|  | Unknown | 3 (1.94%) | 0 (0.00%) |  |
